# Protective effect of extra virgin olive oil (EVOO) consumption on the physical component of health-related quality of life in aging adults

**DOI:** 10.1007/s00394-026-03906-y

**Published:** 2026-02-12

**Authors:** Javier Conde-Pipó, Cristina Molina-Garcia, Julian Arense, José Daniel Jiménez-García, Antonio Martínez-Amat, Miguel Mariscal-Arcas

**Affiliations:** 1 Department of Health Sciences, Faculty of Health Sciences, University of Jaén, Jaén, Spain; 2 Health Science and Nutrition Research (HSNR-CTS1118), Department of Nutrition and Food Science, School of Pharmacy, University of Granada, Granada, Spain; 3 Faculty of Physiotherapy, Podiatry and Occupational Therapy, Catholic University San Antonio-UCAM, 30107 Murcia, Spain; 4 Institute for Biomedical Research of Murcia, IMIB-Arrixaca, 30120 Murcia, Spain; 5 Department of Languages, Arts and Sports, Faculty of Education, University of Málaga, 29009 Málaga, Spain; 6 Instituto de Investigación Biosanitaria de Granada (IBS.GRANADA), Granada, Spain

**Keywords:** EVOO, Mediterranean diet, Health-related quality of life, Bodily pain, Older adult

## Abstract

**Background/Objectives:**

The Mediterranean dietary pattern (MedDiet) is associated with numerous health benefits, particularly in preventing chronic diseases and promoting well-being. Extra virgin olive oil (EVOO), a key component of the MedDiet, is rich in monounsaturated fatty acids (MUFAs), polyphenols, and antioxidants, which may help slow age-related physical decline. Health-related quality of life (HRQoL) is a crucial indicator of population health, and with an aging population, it is essential to assess whether dietary habits influence the physical component (Comp-P) of HRQoL. This study examines this association in older adults to provide evidence supporting dietary recommendations for healthy aging.

**Methods:**

A cross-sectional study was conducted on 180 physically active adults aged 41–80, all adhering to the MedDiet. Data were collected using the MEDAS, RAPA-Q, and SF-36 questionnaires. Participants were classified into two groups based on EVOO consumption: MT4 (≥ 4 tablespoons/day) and LT4 (< 4 tablespoons/day).

**Results:**

In the LT4 group, Comp-P and age showed a moderate, negative, and significant correlation (r = − 0.349, *p* = 0.009), while in the MT4 group, the correlation was weak, negative, and not significant (r = − 0.007, *p* = 0.431). Similarly, bodily pain correlated negatively with age in the LT4 group (r = − 0.328, *p* = 0.014), whereas no significant association was found in the MT4 group (r = 0.102, *p* = 0.234).

**Conclusions:**

Among adults aged 41–80, higher EVOO consumption (≥ 4 tablespoons/day) may have a protective effect, mitigating the impact of aging on self-perceived physical health and functional capacity.

## Introduction

The world’s population is growing rapidly. In 2015, people aged 65 and over accounted for 8.5% of the world’s population, and this proportion is expected to double by 2050 [1]. Increasing life expectancy poses challenges for both society, which needs to adapt to rising healthcare costs and the demands of this demographic shift, and individuals, who need to take preventive measures to maintain independence, well-being and healthy ageing [2].

The goal is therefore not only to live longer, but also to live better, ensuring a higher quality of life in later life. In this context, the World Health Organization (WHO) defines quality of life (QoL) as an individual’s perception of their well-being within the cultural and social context in which they live, considering their goals, expectations, and personal values [3]. A key measure of QoL is health-related quality of life (HRQoL), a multidimensional construct that assesses self-perceived physical and mental health, providing valuable insights at both individual and population levels [4, 5]. Even minor declines in HRQoL can be significant for older adults managing chronic conditions [6]. It is commonly evaluated using self-reported tools such as the SF-36 questionnaire [7].

The physical component (Comp-P) of HRQoL, which includes physical function, physical role, bodily pain and general health status, tends to decline with age due to muscle loss and body composition changes, making it more difficult to perform activities of daily living [2, 8]. Dietary habits play a crucial role in HRQoL, as nutrition directly impacts physical and mental health. While an unhealthy diet can compromise physiological functions and increase the risk of chronic disease, a well-balanced diet strengthens the immune system, supports cognitive function and promotes overall well-being [9].

The Mediterranean dietary patter (MedDiet) is recognised as one of the healthiest dietary models due to its nutritional balance, adequacy, and ease of adherence [10]. It is primarily composed of traditional, often homemade foods and beverages typical of the countries bordering the Mediterranean Sea [11]. The MedDiet is characterised by high consumption of extra-virgin olive oil (EVOO), leafy green vegetables, fruits, cereals, nuts, and legumes, along with moderate consumption of fish, other meats, dairy products, and red wine, and limited intake of eggs and sweets. Its approximate energy content is 2.200 kcal/day, with less than 40% of energy derived from total fat. Additionally, the MedDiet is rich in monounsaturated fatty acids (MUFAs), mainly oleic acid (C18:1n-9, OA), and in polyphenols deriving from EVOO, polyunsaturated fatty acids (PUFAs) from fish, mainly eicosapentaenoic acid (C20:5n-3, EPA) and docosahexaenoic acid (C22:6n-3, DHA); as well as antioxidants and flavonoids contained in fruits, legumes and vegetables [11, 12].

These characteristics contribute to the health benefits of the MedDiet, which is particularly relevant in ageing, as it is associated with increased life expectancy, improved quality of life and reduced risk of developing metabolic, cardiovascular, musculoskeletal and inflammatory diseases [13–15]. In this context, EVOO is considered a nutraceutical and functional food, due to its bioactive compounds which can modulate different processes linked to ageing and age-related diseases associated with chronic low-grade inflammation [16]. To achieve the protective health benefits of the MedDiet, a daily intake of approximately 50 mL (4 tablespoons) of EVOO is recommended [17]. However, EVOO consumption varies even among individuals adhering to the MedDiet, with some studies reporting that around 60% of participants meet the recommendation, while some reporting compliance rates of up to 80% [12]. In Spain, the world’s largest producer of EVOO, the average intake is only 25 mL per day, which is half of the recommended amount [18].

Although evidence suggests a positive association between adherence to the MedDiet and HRQoL, differences in age, health status, lifestyle, and dietary assessment methods contribute to inconsistencies in the results [19–22]. Furthermore, the specific effect of EVOO consumption on HRQoL remains uncertain due to the lack of studies addressing this aspect.

While evaluating overall dietary patterns provides a better understanding of the relationships between diet and health than focusing on individual foods or nutrients [9, 13], we hypothesise that higher EVOO consumption, even among those adhering to the MedDiet, may contribute to mitigating the effects of ageing and improving HRQoL.

The present study aimed to examine the association between extra virgin olive oil (EVOO) consumption and the physical component of health-related quality of life (HRQoL) in physically active middle-aged and older adults adhering to the Mediterranean diet, to determine whether higher EVOO intake contributes to preserving functional capacity and well-being with aging.

## Material and methods

### Participants and study design

The study followed a cross-sectional, descriptive and comparative design. Four inclusion criteria were defined: (a) being between 41 and 80 years of age, (b) having an active lifestyle according to the WHO physical activity guidelines, (c) being in good health with no conditions that interfere with daily activities, and (d) having a good adherence to the Mediterranean dietary pattern. Of the initial 553 participants recruited from different regions of Spain, 373 were excluded because they did not meet the inclusion criteria or did not complete the questionnaires correctly. Therefore, the final sample consisted of 180 participants. Recruitment was carried out randomly over a 3-month period and participants were invited to take part in the study on a voluntary basis after giving informed consent. They were given detailed information about the aims of the study and were assured of the strict confidentiality of their data. The study adhered to the ethical principles of the Declaration of Helsinki and was approved by the Research Ethics Committee of the University of Granada.

### Instruments

All participants completed the following questionnaires, either for the selection of the final sample or for subsequent analysis of the variables of interest.

#### Sociodemographic and health data

Sociodemographic information and health status were collected using an ad hoc questionnaire, which included age, gender, weight, height, and any diseases experienced in the last 12 months, such as cardiovascular disease (CVD), cholesterol or diabetes.

#### Physical activity

Physical activity (PA) was assessed using the Spanish version of the Rapid Assessment of Physical Activity (RAPA-Q) [23], a validated and user-friendly tool specifically designed for older adults. This seven-item questionnaire has “yes” or “no” responses and effectively determines physical activity levels. Following the WHO recommendations for cardiovascular health benefits [24], participants engaging in more than 150 min per week of moderate activity or 75 min of vigorous activity were classified as active.

#### Adherence to the Mediterranean diet pattern and olive oil consumption

The questionnaire Mediterranean Diet Adherence Screener (MEDAS) [25] was used to quantitatively assess adherence to the MedDiet. This validated tool, derived from a food frequency questionnaire (FFQ, r = 0.52; *p* < 0.001), was specifically developed to assess the role of the MedDiet in the primary prevention of cardiovascular diseases [25–28]. The MEDAS comprises 14 questions, 12 of which focus on the intake frequency of key dietary components, including EVOO, wine, fruits, vegetables, fish, legumes, nuts, meat and its derivatives, poultry, butter, pastries, and carbonated/sweetened beverages. The remaining two questions examine specific characteristics of the MedDiet, such as the exclusive use of EVOO for cooking and the consumption of chicken. Each affirmative response contributes one point to the total score, with values of 10 or higher indicating good adherence to the MedDiet, while scores below 10 indicate poor adherence [29]. Participants categorised as poor adherence were excluded in the study. A positive response to the question on EVOO consumption (“How much do you consume daily, including for frying, salads, meals eaten away from home, etc.?”) indicated a daily intake of ≥ 4 tablespoons (1 tablespoon = 13.5 g). Based on this, the final sample were classified into two groups: LT4 (less than 4 tablespoons) and MT4 (4 or more tablespoons).

#### Health-related quality of life

The Spanish version of the SF-36 questionnaire [30] was used to evaluate health-related quality of life (HRQoL). This instrument has been widely validated, demonstrating high reliability and extensive use in older adult populations [31–33]. It comprises eight domains: physical function (10 items, α = 0.93), physical role (4 items, α = 0.95), bodily pain (2 items, α = 0.87), general health (5 items, α = 0. 79), vitality (4 items, α = 0.85), social function (2 items, α = 0.72), emotion-al role (3 items, α = 0.91) and mental health (5 items, α = 0.85). Each domain is scored on a scale from 0 to 100, with higher scores indicating better health status. Additionally, the SF-36 provides two summary scores: the physical component (Comp-P) and the mental component (Comp-M), which are calculated using population-specific weights for Spain [34]. The cut-off points for classifying these components into two levels were based on median values from Spanish population norms, adjusted by age and gender [35]. In this study, the Cronbach’s α coefficients for the SF-36 exceeded 0.75, indicating satisfactory internal consistency.

### Statistical analysis

Statistical analyses were conducted using R statistical software (R Core Team, Vienna, Austria). The Kolmogorov–Smirnov test with Lilliefors correction was applied to assess the normality of the variables, while Levene’s test was applied to evaluate homoscedasticity. Descriptive statistics are presented as means ± standard deviations (SD) and frequencies (%) for categorical variables. Group comparisons for continuous variables were conducted using the non-parametric Mann–Whitney U test, given that the assumptions for a parametric t-test were not met. The Pearson Chi-square test was used for categorical variables comparisons. Bivariate correlations were assessed using Spearman’s rho correlation coefficient. The internal reliability of the instruments was evaluated using Cronbach’s Alpha. All *p* values were two-tailed, with statistical significance set at *p* ≤ 0.05.

## Results

A total of 180 participants met the inclusion criteria and were subsequently evaluated. Their anthropometric characteristics and health status, stratified by sex and EVOO consumption groups (LT4 and MT4) are presented in Table 1. Significant sex-based differences were observed in several anthropometric variables, including height, weight, body mass index (BMI), as well as in the prevalence of diabetes and musculoskeletal disease, which were higher in men (*p* ≤ 0.05). However, when comparing LT4 vs. MT4, no statistically significant differences were found in anthropometric measures, physical component scores, or health conditions.

**Table 1:** Anthropometric characteristics and health status by sex and Olive oil consumption group

Variables	Sample			Group		
	M	W	*p*_sex_	LT4	MT4	*p*_group_
Distribution, n, %	131, 72.78	49, 27.22	0.001	55, 30.56	125, 69.44	0.001
Age, years*	57.38 (7.48)	55.38 (9.74)	0.099	55.52 (8.02)	57.41 (8.21)	0.301
Height, m*	1.75 (6.85)	1.60 (7.60)	0.001	1.73 (8.29)	1.71 (10.02)	0.359
Weight, kg*	79.18 (9.64)	62.64 (13.07)	0.001	76.04 (12.44)	74.33 (13.07)	0.440
BMI, kg/m^2^*	25.70 (24.23)	2.99 (4.89)	0.001	25.27 (3.00)	25.33 (3.88)	0.939
Physical component score*	50.41 (7.08)	52.51 (7.16)	0.172	51.50 (6.61)	50.76 (7.38)	0.798
Hypertension (yes), %	15.45	12.82	0.739	13.33	15.38	0.941
Diabetes (yes), %	7.27	0.00	0.018	4.44	5.77	1.000
Hypercholesterolemia (yes), %	17.27	23.08	0.394	11.11	22.12	0.179
Cardiovascular disease (yes), %	2.73	0.00	0.182	2.22	1.92	1.000
Musculoskeletal disease (yes), %	7.27	0.00	0.017	8.89	3.85	0.388
Respiratory disease (yes), %	6.67	4.81	0.121	6.675	4.81	0.953

*Mean (SD); M: man; W: woman

**Fig. 1:**
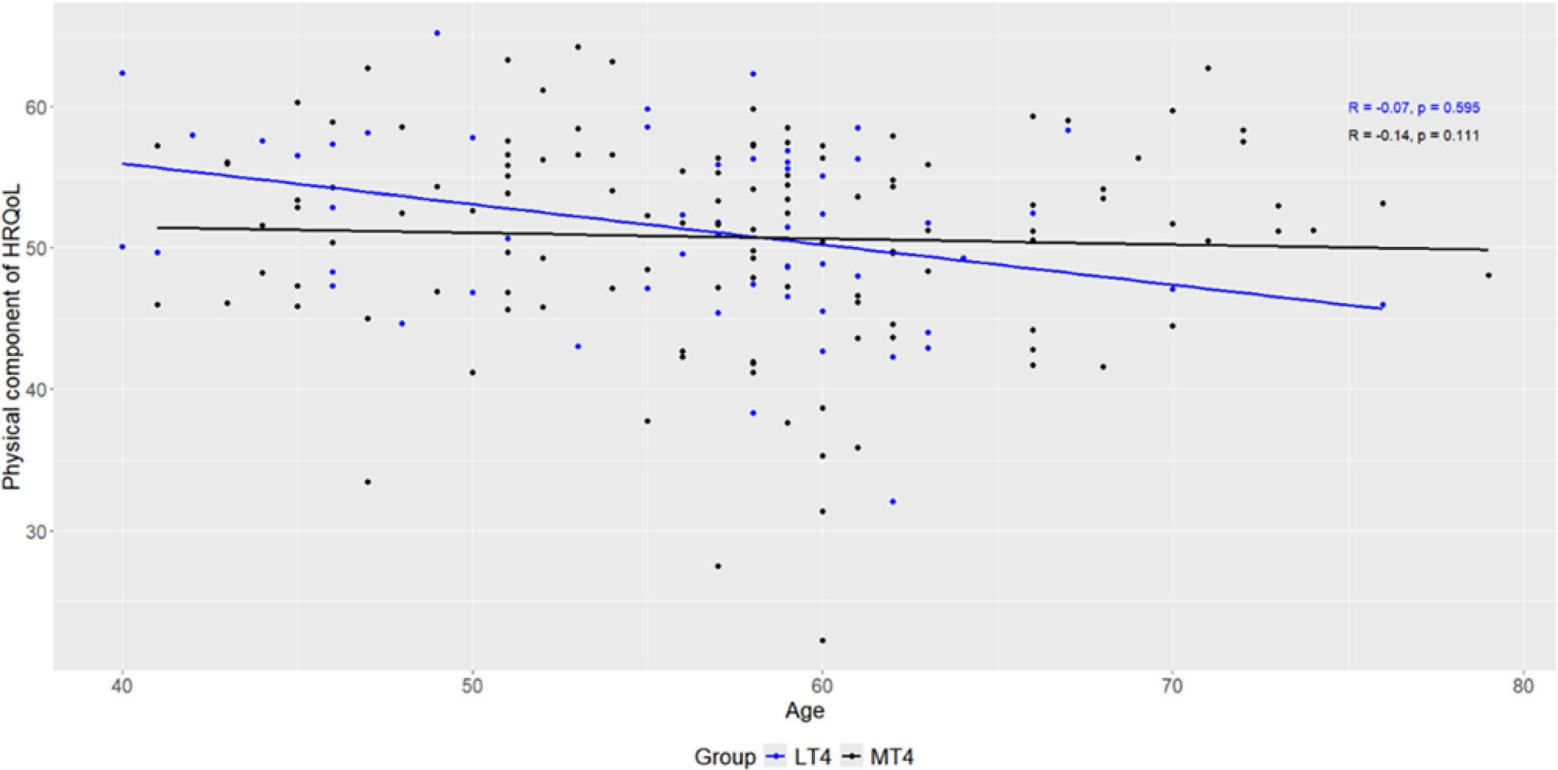
Association between age and Comp-P by EVOO consumption groups

Table 2 and Fig. 1 shows the bivariate correlations between age and the physical component (Comp-P) and its dimensions, stratified by EVOO consumption groups. A significant negative correlation was observed between the overall Comp-P score and age in the LT4 group (r = − 0.35, *p* = 0.009), whereas no significant association was observed in the MT4 group (*p* = 0.431). Among the Comp-P dimensions, physical functioning showed a significant negative correlation with age in both groups (LT4: r = − 0.39, *p* = 0.003; MT4: r = − 0.37, *p* = 0.001). Notably, bodily pain was also significantly correlated with age in the LT4 group (r = − 0.33, *p* = 0.014), but this association was not observed in the MT4 group (*p* = 0.234), suggesting a potential moderating effect of higher EVOO consumption. No significant correlations were found for role physical or general health in either group (*p* > 0.05).

**Table 2:** Bivariate correlation of physical dimensions of the SF-36 with age by group

SF-36 dimension	LT4			MT4		
	r	CI	p*	r	CI	p*
Physical functioning	− 0.39	(− 0.58, − 0.17)	**0.003**	− 0.37	(− 0.52, − 0.19)	**0.001**
Role physical	− 0.07	(− 0.32, 0.20)	0.829	− 0.14	(− 0.31, 0.01)	0.111
Bodily pain	− 0.33	(− 0.52, − 0.11)	**0.014**	0.10	(− 0.07, 0.26)	0.234
General health	0.03	(− 0.20, 0.27)	0.829	− 0.12	(− 0.31, 0.05)	0.181
Physical component Comp-P)	− 0.35	(− 0.58, − 0.09)	**0.009**	− 0.07	(− 0.24, 0.10)	0.431

*Values in bold indicate statistically significant correlations (*p* ≤ 0.05)

## Discussion

This study explored the relationship between extra virgin olive oil (EVOO) consumption and the physical component (Comp-P) of health-related quality of life (HRQoL) in physically active middle-aged and older adults adhering to the Mediterranean dietary pattern (MedDiet). To our knowledge, this is the first study to specifically assess this association.

Our findings indicate that in individuals with higher EVOO consumption (≥ 4 tablespoons per day), neither the Comp-P nor the bodily pain HRQoL domains showed a significant correlation with aging, maintaining a stable trend. In contrast, those with lower EVOO intake showed a significant negative association, suggesting a decline in Comp-P and an increase in bodily pain with age. These results suggest that higher EVOO consumption may have a protective effect on HRQoL, potentially mitigating age-related declines in functional capacity and pain perception.

It should be noted that the SF-36 bodily pain subscale reflects overall pain perception and does not differentiate between musculoskeletal and other pain sources, which may be influenced by comorbid conditions common in older adults. This consideration is particularly relevant when interpreting pain-related findings in aging populations, as pain of different origin, such as articular, neuropathic, or inflammatory, may coexist and vary in intensity. Consequently, the observed stability of pain perception in the higher EVOO consumption group may reflect not only a lower inflammatory burden but also a broader modulation of perceived discomfort associated with aging.

These findings should be interpreted within the broader context of nutritional challenges faced by older adults. Aging populations often exhibit reduced intake of high-biological-value proteins, vitamins, and natural antioxidants, which can exacerbate sarcopenia, oxidative stress, and functional decline. Therefore, maintaining an adequate and high-quality diet, such as the Mediterranean pattern enriched with EVOO, may help counteract these processes and preserve physical function.

To minimise potential confounding, PA and adherence to the MedDiet were included as inclusion criteria, as both are well-documented contributors to improved HRQoL [36]. By controlling these variables, our findings more accurately reflect the association between higher EVOO intake and the preservation of Comp-P and pain reduction in aging.

Our results are consistent with previous studies in the biomedical field. A growing body of preclinical, population, and clinical studies suggests that adherence to the MedDiet, rich in biophenols, may help prevent metabolic disorders and age-related decline by regulating redox balance and inflammation [37]. Although biophenols present in key MedDiet foods, mainly in EVOO, have low bioavailability, their continuous intake over a lifetime that is associated with a protective effect against age-related diseases [38].

While the precise molecular mechanisms of EVOO´s effects are not fully understood, its health benefits are largely attributed to its bioactive components, mainly MUFAs, PUFAs, tocopherols and polyphenols, with biophenols being the most abundant [38]. Among these, the phenolic compound oleocanthal, shares the same anti-inflammatory mechanism as ibuprofen by inhibiting cyclooxygenase (COX) enzymes, which regulate inflammation and pain [39, 40]. The beneficial effects of EVOO in aging may be attributed not only to its anti-inflammatory action but also to its role in oxidative stress regulation. By improving redox balance, EVOO could help restore neuronal function, potentially impacting cognitive health, neurodegenerative diseases and overall HRQoL in older adults [37].

The medicinal potential of natural polyphenols has led to extensive research in cell culture, animal models and human trials [37]. In animal models, Fezai et al. [41] reported that EVOO showed significant antinociceptive, anti-inflammatory, and anti-cancer properties. Also, Szychlinska et al. [42] and Chiang et al. [40] applied EVOO treatment, and observed a significant cartilage recovery process in early osteoarthritis and lower abdominal pain, respectively.

In human studies, although research into the relationship between EVOO and pain remains limited, some findings suggest its potential benefits. A pilot study in patients with trigeminal neuralgia found that consuming 60 mL of EVOO daily for 12 weeks significantly reduced pain interference with daily activities and improved quality of life, as measured by the SF-36 questionnaire [43]. Similarly, a study in fibromyalgia patients reported that consumption of 50 mL of EVOO daily for 3 weeks led to a significant reduction in inflammation and cardiovascular risk markers, further supporting its antithrombotic and anti-inflammatory effects [44]. Additionally, a randomized clinical trial in individuals with severe obesity found that EVOO consumption, alone or with a traditional Brazilian diet, significantly reduced severe and musculoskeletal pain [45]. Moreover, recent research on rheumatoid arthritis patients suggests that EVOO polyphenol-enriched extracts may exert strong anti-inflammatory and antioxidant effects, helping reduce pro-inflammatory cytokines (TNF-α and IL-1β) and oxidative stress markers [46].

These findings highlight the potential role of EVOO in inflammatory and pain-related conditions and align with our results suggesting that EVOO may contribute to pain management and functional well-being. All these studies corroborate, in the short term, what we hypothesized and observed in the long term: higher EVOO consumption may play a protective role in aging by preserving physical component of HRQoL and reducing pain-related impairments. Beyond its MUFA content, EVOO contains key phenolic compounds such as hydroxytyrosol, which exert strong antioxidant and anti-inflammatory effects. Hydroxytyrosol has been associated with improved endothelial function, lipid metabolism, and neuroprotection, contributing to reduced risk of cardiovascular, hepatic, and neurodegenerative diseases [47, 48]. These findings reinforce the potential of EVOO as a functional food that promotes healthy aging.

### Strengths and limitations

This study has several strengths. To our knowledge, it is the first to specifically examine the association between EVOO consumption and the physical component (Comp-P) of HRQoL, contributing novel insights to the existing literature. The inclusion of physically active middle-aged and older adults adhering to the MedDiet allowed us to control for key confounders, ensuring that the observed associations were not influenced by broader dietary patterns or physical activity levels. Additionally, HRQoL was assessed using the SF-36 questionnaire, a widely validated tool that provides a comprehensive evaluation of self-perceived physical function and pain.

However, some limitations should be considered. First, the cross-sectional design prevents establishing causality between EVOO consumption and HRQoL outcomes. Longitudinal or intervention studies are needed to confirm whether higher EVOO intake directly contributes to better physical function and lower pain levels over time. Second, self-reported dietary data may be subject to recall bias or misreporting, potentially affecting the accuracy of EVOO intake estimations. EVOO consumption was self-reported through the MEDAS questionnaire, which may be subject to recall bias or under-/overestimation of intake, potentially affecting accuracy in the classification of consumption levels. Third, while participants followed the MedDiet, variations in total dietary intake and nutrient composition were not controlled, which could have influenced the results. Finally, the sample was limited to physically active individuals, which may not fully represent the general aging population, particularly those with lower activity levels or pre-existing health conditions. Therefore, the generalizability of these findings should be interpreted with caution, as the protective association observed with higher EVOO consumption may differ in less active or metabolically compromised older adults.

Future research should focus on longitudinal studies and randomized controlled trials to further explore the impact of EVOO consumption on HRQoL, functional capacity, and pain management, particularly in diverse populations with varying levels of physical activity and dietary adherence.

## Conclusions

This study provides novel evidence suggesting that higher EVOO consumption is associated with the preservation of physical function and reduced pain perception in aging. Among physically active middle-aged and older adults adhering to the MedDiet, those with higher EVOO intake (≥ 4 tablespoons/day) showed no significant correlation between aging and declines in the physical component of HRQoL, while those with lower consumption exhibited a significant negative association. Although research on the relationship between EVOO and HRQoL remains limited, our findings align with previous studies emphasizing the anti-inflammatory and antioxidant properties of EVOO, which may contribute to better functional capacity and overall well-being.

Promoting regular EVOO consumption within the Mediterranean diet could represent a simple and sustainable public health strategy to support healthy aging, aligning with current efforts to extend both life expectancy and years lived in good health and functional independence.

## Data Availability

There are restrictions on the availability of the data for this trial due to the signed consent agreements around data sharing, which only allow access to external researchers for studies following the project’s purposes. Requestors wishing to access the trial data used in this study can make a request to mariscal@ugr.es.
